# Are Frail Older People from Racial/Ethnic Minorities at Double Jeopardy of Putting off Healthcare during the Pandemic?

**DOI:** 10.3390/ijerph20021034

**Published:** 2023-01-06

**Authors:** Dongjuan Xu, Greg Arling

**Affiliations:** School of Nursing, Purdue University, West Lafayette, IN 47907, USA

**Keywords:** frailty, race/ethnicity, putting off healthcare, older adults, COVID

## Abstract

Given the differential impacts of COVID-19 on racial and ethnic groups, it is unclear how racial/ethnic status and frailty combine to influence pandemic-related healthcare disruptions. This study aimed to test the double jeopardy hypothesis: racial/ethnic minority older adults suffer a double disadvantage in access to health care during the pandemic due to the interactive effects of frailty and race. This study uses the linked National Health and Aging Trends Study (NHATS) and COVID-19 public use data files. A multivariate logistic regression model was performed. Overall, approximately two out of five (41%) older adults reported postponing care due to the pandemic. The likelihood of putting off care increased slightly by frailty status. We found no significant difference between Whites and non-Whites in putting off care. However, the simple comparison masked significant variation across frailty status. Robust non-White older people were less likely to put off care than robust Whites (robust non-Whites: 29% vs. robust Whites: 39%); in contrast, frail non-White older people were more likely to put off care (frail non-Whites: 55% vs. frail Whites: 42%). Being frail and non-White creates double jeopardy, which has a negative impact on access to healthcare. Timely access to care is essential for frail older people, particularly non-Whites, because of their complex health conditions accentuated by health and social disparities.

## 1. Introduction

The concept of double jeopardy reflects the combined negative impacts of long-standing race- and age-based inequities and social vulnerabilities on older Black adults [1,2]. The basic idea is that the combined effect of two disadvantages may be greater than the sum of the two individually, which is also referred to as intersectionality. Frailty was defined as “increased vulnerability to stressors as a result of aging and the accumulation of multiple underlying physiological declines” [3,4]. Frailty along with advanced age-associated chronic conditions greatly increases the risk that a person will suffer severe symptoms or death from COVID-19 [5]. Racial and ethnic minorities have suffered disproportionately high rates of COVID-19 incidence, hospitalization, and mortality [2,6,7,8,9]. An array of factors has been speculated to account for these racial and ethnic disparities, including differences in biology, preexisting underlying health conditions, employment-related risk for infection, and “weathering processes” [2,8,10,11]. Frail older people benefit from continuity of care in order to manage their chronic conditions [12,13]. However, using the health care system along with other outside activities can increase exposure to COVID-19. Fear of exposure may contribute to putting off care. It is unclear how the situation of double jeopardy—being both frail and non-White—shaped older adults’ care seeking behaviors and influenced their continuity of care during the pandemic. 

In the U.S., there is no single healthcare system. Health insurance is provided through a mixture of public and private programs. Medicare, a federal health insurance program for older adults, provides health coverage with premium contributions and cost-sharing. Medicaid provides long-term care for older adults with low incomes and resources. Medicaid eligibility and benefits vary considerably from State to State. Major barriers to health care include financial barriers such as high costs and inadequate insurance coverage, structural barriers such as lack of access to services, lack of resource availability, and transportation difficulty, interpersonal barriers such as patient-provider relationship, language barriers, and mistrust of the healthcare system, and personal barriers such as health literacy, and religious and cultural beliefs [14,15,16,17]. Prior studies have found racial and ethnic disparities in access to and quality of care [17,18]. Black and Hispanic adults are less likely to receive and to be able to carry out treatments for heart disease [19], cancer [20], hypertension [21,22], diabetes [23], and dyslipidemia [24], as their White counterparts. Racial and ethnic disparities also exist in screening and preventive services, newer therapies, and invasive procedures [25,26]. The root cause of these healthcare disparities can be linked to effects of institutional racism. Racial and ethnic disparities in access to and quality of health care are only partially explained by differences in socioeconomic status, health insurance coverage, or other factors [8,18,21], although it is difficult to separate the contributions of race/ethnicity and socioeconomic status [27] and fully partial out the influence of socioeconomic status [28]. Implicit racial/ethnic bias—negative attitudes toward people of color and positive attitudes toward Whites—has been recognized in health care settings [29,30]. Prior studies have found that perceived racial bias and discrimination were related to delays in seeking healthcare including delaying filling prescriptions, delaying tests or treatments, and poor adherence to care recommendations [31,32]. Implicit bias combined with poor prior patient-provider interactions may lead minorities to avoid seeking healthcare until their symptoms are life-threatening [6,33]. Race-concordant visits have been characterized by more favorable communications and satisfaction with their health care provider, contributing to continuity of care [34,35]. About 22% of African Americans preferred an African American physician and one-third of Latinos preferred a Latino physician [35]. Black and White older people are to a large extent treated by different primary care providers and providers treating Black older people reported facing greater difficulties in obtaining access to important clinical resources than those treating White counterparts [36]. Lack of English proficiency also may limit awareness and understanding of the available options for prevention and management of chronic conditions [17,37]. 

The impact of COVID-19 on older adults goes beyond a higher risk for serious symptoms. Compared with those in other high-income countries, older adults in the U.S. were the most likely to have economic hardships related to the pandemic, to have appointments cancelled or postponed because of the pandemic, and to report not receiving help such as housework, meal preparation, shopping, or medication management,—either from informal caregivers like family or friends or from professional caregivers—because services were cancelled or very limited during the pandemic [38]. Such impact may be exacerbated further for racial and ethnic minorities. Black and Latino/Hispanic older adults were far more likely than White older adults (White: 14%, Black: 32%, and Latino/Hispanic: 39%) to experience economic difficulties with more losing a job or using up all or most of their savings during the pandemic [38]. Racial and ethnic minorities make up a large part of the essential workforce [7]. Relative to Whites, racial and ethnic minorities are more likely to live in multigenerational households and households containing health-sector workers [10,39]. Compared with White adults at high risk for severe illness, a larger share of Black and Hispanic adults lived in households with at least one worker who was unable to work from home (White: 46.6%, Black: 56.5%, and Hispanic: 64.5%) [10]. In contrast to Whites, racial and ethnic minorities had lower knowledge of COVID-19 and poorer attitudes toward COVID-19, but better practice to reduce risk of COVID-19 [40]. Given the differential impacts of COVID-19 on racial and ethnic groups, it is unclear whether there are racial/ethnic differences in pandemic-related disruptions in their care, and whether being frail and non-Whites together creates a double jeopardy for older adults during the pandemic. Reducing racial and ethnic disparities in access to and quality of care is an essential component to diminish health disparities. 

Using a nationally representative sample of Medicare beneficiaries aged 65+, this study aims to explore the concept of “double jeopardy” and answer a series of research questions: (1) Does frailty increase the likelihood that older people will put off healthcare during the COVID-19 pandemic? (2) Are older people from racial/ethnic minorities more likely to put off care than White older people? (3) Do racial disparities and frailty status put older, frail Blacks and other ethnic groups at double jeopardy of putting off care? That is, do race/ethnicity and frailty status each have an additive effect on putting off care? Alternatively, might frailty status moderate the relationship between race/ethnicity and putting off care, with frailty exacerbating other racial/ethnic disparities and contributing to greater putting off care for frail Black and other minorities compared with frail Whites? and (4) Are there differences by race/ethnicity and frailty status in the reasons given by older people for putting off care?

## 2. Materials and Methods

### 2.1. Data and Sample

This study uses the linked National Health and Aging Trends Study (NHATS) Round 9 and COVID-19 public use data files. The NHATS is a longitudinal study that annually surveys a nationally representative sample of Medicare beneficiaries ages 65 or older. The COVID-19 data file is a supplement to the NHATS and collected information about participants’ experiences during the COVID-19 outbreak from June 2020 to January 2021. This study included community-dwelling older adults who responded to the COVID survey on whether they put off their healthcare during the COVID-19 pandemic (*n* = 2986). Exclusion criteria included (1) living in a residential care setting (*n* = 214, 7.2%) and (2) having missing data for some of the study variables including education, health conditions, and others help with medicines (*n* = 144, 4.8%). Hence, our final sample size totaled 2628 older adults and the weighted population sample size was 22,164,441.

### 2.2. Measures

#### 2.2.1. Dependent Variable

**Putting off care**. Participants were asked, “During the COVID-19 outbreak, has there ever been a time when you needed or had planned to see a doctor or other health care provider but put off getting care?” For those with an affirmative response, participants were followed with the types of care they put off and reasons for putting off care. The following reasons were listed: (1) I couldn’t afford it, (2) I couldn’t get an appointment, (3) The provider cancelled, closed, or suggested rescheduling, (4) I decided it could wait, (5) I was afraid to go, (6) A family member did not want me to go, and (7) other reason. Participants were asked to choose all the reasons that applied. Unfortunately, the survey did not contain direct measures of system-level racial/ethnic biases or disparities.

#### 2.2.2. Independent Variables

**Frailty**. Physical frailty phenotype was assessed using five criteria based on nationally representative standards: low physical activity, slowness, weakness, exhaustion, and shrinking [41]. Participants who met three to five criteria were considered “frail”, those who met one to two criteria were “pre-frail”, and those with none were “robust”. 

**Race/ethnicity**. Participants were first grouped into three categories: White, non-Hispanic; Black, non-Hispanic; and Hispanic or other racial or ethnic group. Due to the very small sample size in the “robust other races group” (*n* = 25), we used two race/ethnicity groups in the analysis: White, non-Hispanic (“White”) and Black, Hispanic and other racial or ethnic groups (“non-White”). 

#### 2.2.3. Control Variables

In addition to the aforementioned variables, analyses included categorical age (65−79 and 80+ years), sex (male and female), marital status (married/partnered or unmarried), education (less than high school, high school or associate’s degree, and bachelor’s degree or above), and location (metropolitan and non-metropolitan). We counted the number of high-risk health conditions for COVID-19 complications. The high-risk conditions consisted of lung disease, heart attack or myocardial infarction, heart disease, high blood pressure, stroke, diabetes, dementia or Alzheimer’s disease, and cancer. We further categorized the number of high-risk conditions into two groups: fewer than 3 conditions and 3+ conditions. Participants were also asked whether someone helped keep track of their prescriptions during the COVID-19 outbreak. Finally, participants in the pre-COVID-19 Round 9 survey were asked if they had a regular doctor and if they saw the doctor in the prior year. 

### 2.3. Statistical Analysis

Descriptive statistics were used to describe the characteristics of participants. To explore whether older people with frailty and from racial/ethnic minorities were more likely to put off care during the COVID-19 pandemic, a cross-tabulation analysis was conducted between frailty and race/ethnicity regarding putting off care. To investigate whether frailty status modified the relationship between race/ethnicity and putting off care, a multivariate logistic regression model with an interaction term between frailty and race/ethnicity was performed. The logistic regression model was adjusted for age, sex, marital status, education level, location, high-risk conditions, and whether others helped with prescriptions. A figure was plotted to depict the predicted probability of putting off care by frailty and race/ethnicity groups. Moreover, we described the types of care older people put off and reasons for putting off care. Regarding reasons for putting off care, since participants were allowed to respond to more than one reason, we conducted corrected chi-squared tests [42]. Guidelines regarding survey weights, strata and cluster were used to conduct the weighted analysis and adjust standard errors and statistical tests to account for the complex design of NHATS [43]. All analyses were conducted using Stata Version 16.1 (StataCorp, College Station, TX, USA). Statistical significance was accepted at the *p* < 0.05 (two-sided) level.

## 3. Results

Population-weighted characteristics of study participants are presented in Table 1. Approximately 27% of older people were 80 years or older and more than half (54%) were female. Roughly 19% lived in a non-metropolitan area and 18% had three or more high-risk conditions for COVID-19 complications. The majority (85%) was White, non-Hispanic. The largest percentage of older people was pre-frail (71%); about equal percentages were robust (15%) or frail (14%). Nearly 41% reported putting off healthcare when needed or had planned to see a health care provider during the COVID-19 outbreak. During the pre-COVID period in 2019, a very high percentage of participants reported having a regular doctor (97%) and seeing the doctor during the year (95%).

**Figure 1:**
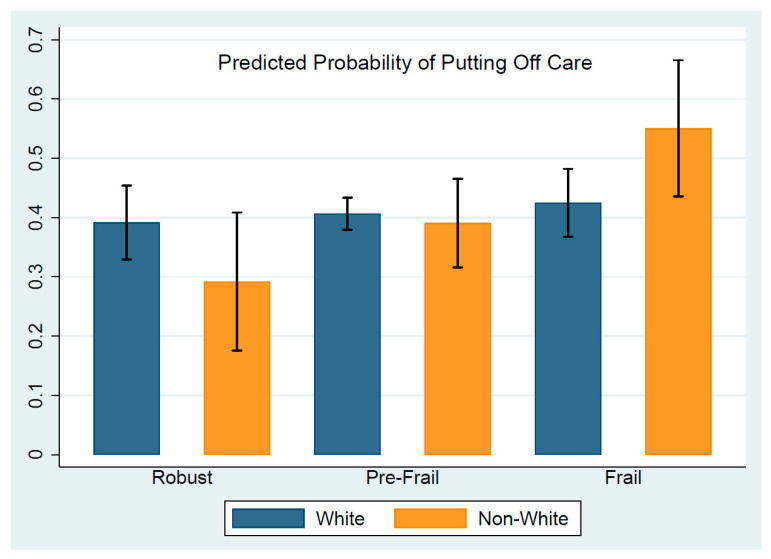
Predicted probability of putting off care by frailty and race/ethnicity.

As shown in Table 2, the percentage of putting off care increased slightly by frailty status (robust: 38%, pre-frail: 41% and frail: 41%); however, the difference was not statistically significant. Older people from racial/ethnic minorities had similar percentage of putting off care as White older people (non-Whites: 38%; Whites: 41%). For White, non-Hispanic older people, putting off care did not vary by frailty status. Similar percentages of White, non-Hispanic older people reported putting off care among the robust (40%), pre-frail (42%) and frail (39%) groups. For non-White older people, putting off care increased with frailty status: 29% for robust, 38% for pre-frail, and 51% for frail. The findings suggest that frailty status modified the relationship between race/ethnicity and putting off care. The logistic regression model, controlling for sociodemographic and health-related variables, tended to confirm the modifying role of frailty status (Table 3). The interaction term between frail status and racial/ethnic minorities was statistically significant (*p* = 0.009, Table 3). As illustrated in Figure 1, the predicted probability of putting off care remained the same among White, non-Hispanic older people, regardless of frailty status. In contrast, frailty increased the likelihood that non-White older people put off healthcare during the COVID-19 pandemic. Robust non-White older people were less likely to put off care than robust Whites (robust non-Whites: 29% vs. robust Whites: 39%), while in contrast, frail non-White older people were more likely to put off care than frail Whites (frail non-Whites: 55% vs. frail Whites: 42%). Our findings also indicated that older people who were female (OR = 1.7, *p* < 0.001), had a bachelor’s degree or above (OR = 2.3, *p* < 0.001), or lived in a metropolitan area (OR = 1.3, *p* = 0.047) were more likely to put off care during the COVID-19 pandemic (Table 3).

Regarding types of care, dentist or hygienist appointments (55%), seeing a usual doctor (48%), seeing a specialist (40%), and vision appointments (34%) were more likely to be put off during the COVID-19 pandemic. The most common reason for putting off care was providers cancelled, closed, or suggested rescheduling (57%). This was followed by multiple reasons related to the respondent including, decided to wait (52%), was afraid to go (28%), could not get an appointment (11%), a family member did not want him or her to go (8%), and could not afford it (2%). There were no statistically significant differences in reasons for putting off care by race/ethnicity and frailty status (Table 4).

## 4. Discussion

Using a nationally representative sample of community-dwelling older people aged 65 years or older, this study examined the concept of double jeopardy—being both frail and non-White—and its impact on healthcare disruptions during the COVID-19 pandemic. The study found about 41% of older adults put off needed or planned care during the pandemic. Overall, the likelihood of putting off care increased slightly by frailty status, although the difference was not statistically significant. Although frail older people may have had an increased risk of COVID-19 exposure by entering a healthcare setting, they were also most in need of care continuity. Overall, we found no significant difference between Whites and non-Whites in their reports of putting off care. However, the simple bi-variate comparison masked significant variation across frailty status. Robust non-White older people were less likely to put off care than robust Whites; while in contrast, frail non-White older people were more likely to put off care. Our findings highlight the important role frailty status played in racial/ethnic differences in putting off care during the COVID-19 outbreak. Being frail and non-White creates a double jeopardy, which exaggerates disparities in access to healthcare. Timely access to care is very important for frail older people, particularly non-Whites, because effective and good care requires coordination and follow-up plans. There is a clear need to further reduce racial and ethnic disparities in access to and quality of care, which is increasingly a priority for policymakers and health care systems.

The greater putting off of care for frail older Blacks, Hispanics, and other racial/ethnic groups may be the result of systematic barriers to care perhaps heightened during the pandemic. Low health literacy may play an important role, which is a barrier to effective care by limiting older people’s understanding of health information and awareness of care needs. There was differential prevalence of inadequate or marginal health literacy among race/language groups (52.1% of Blacks, 34.3% of Spanish-speaking Hispanics, 29.5% of English-speaking Hispanics, and 18.9% of Whites) [44]. Racial and ethnic minorities had lower levels of knowledge and attitudes related to COVID-19 than Whites [40]. However, findings from the survey suggest that systemic barriers may not have played a major role in putting off care for frail non-Whites. Compared to White older people, non-White older people were no more likely to report affordability as a reason for putting off care. Moreover, non-White older people of all frailty statuses were no more likely than White older people to report that a provider cancelled, closed, or suggested rescheduling, deciding it could wait, being afraid to go, not getting an appointment, and a family member not wanting them to go (Table 4). Nonetheless, older racial/ethnic group members may have experienced other barriers. Frail non-White people who put off care were more likely to choose the “other reason” response. Among those other reasons could have distrust of providers or perceived biases toward racial and ethnic minorities. A 2020 survey found that Black adults reported experiencing discrimination and unfair judgment in healthcare at a rate almost three times higher than White adults and about twice as high as Latino/Hispanic adults [45]. The embedded racism in the healthcare system and the historically long-standing distrust may deter racial and ethnic minorities from seeking routine care, confiding in their providers, and adhering to healthcare recommendations [46,47,48]. This racism and distrust, in addition to factors such as lack of access to information, low health literacy, and racial, cultural, and linguistic incongruence with health care providers may affect frail racial/ethnic minorities in seeking treatment, which further perpetuates racial disparities in access to and quality of health care.

The COVID-19 pandemic has also limited formal and informal supportive services for older people with two or more chronic conditions [38]. Frail older adults needed more support from family members or friends for care-seeking activities such as giving a ride, helping on an exam table, dressing and undressing, reminding about things to ask or tell the doctor, and helping on communication with the doctor before the pandemic in 2019 (Table S1). Older people from the racial/ethnic minority groups needed such help more than their White counterparts. The availability of caregivers was limited and disrupted during the COVID-19 pandemic; therefore, frail older people were not able to receive help they needed to see a doctor. The problem may be more severe among the frail minorities, contributing to a greater likelihood of putting off care.

Older females and those who were living in a metropolitan area or with a college degree were also more likely to put off care. In normal times, in the absence of a pandemic, these characteristics have been associated with care seeking [49,50,51]. A study of gender differences in COVID-19 attitude and behavior found that women were more likely to perceive the pandemic as a very serious health problem, and to agree and comply with restraining public policy measures such as keeping social distancing, staying at home, avoiding crowded places, and stopping meeting friends, after controlling for sociodemographic characteristics and employment status [52]. Another study found that there were no gender differences in knowledge and attitudes related to COVID-19, but women tended to be more likely to report engaging in better practices to reduce their risk of spread and infection with COVID-19 [40]. The greater putting off of care for older adults living in a metropolitan area during the pandemic may partly indicate their less limited access to telemedicine [53]. Previous studies suggested that people with higher educational attainment were less likely to use doctor consultation [49], family physician and hospital services, but more likely to use specialist services than those with fewer years of education [54]. The less intensive use of general practitioners by people with higher educational attainment may be partly explained by systematic differences in socioeconomic position regarding interpretation of symptoms and perception of the need for health care [49]. This may be also reflected in our finding that older adults with a college degree were more likely to put off care during the pandemic. Putting off care to reduce exposure to COVID-19 may be viewed as a sensible option.

This study has several limitations. First, the NHATS sample and the COVID special sample in particular may be unrepresentative of the older minority population. Due to the small sample sizes of Hispanic, Asian, American Indian, or other racial or ethnic groups, we can only explore racial and ethnic disparities in putting off care between Whites and non-Whites. Future data collection efforts may consider further oversampling minority groups. Second, direct measures of system-level racial/ethnic biases or disparities are not available. Third, there are no survey questions pertaining to racism or ethnic biases as a reason for postponing care. More information would be beneficial for further examining the racial and ethnical disparities in putting off care during the pandemic. Fourth, regarding the putting off care questions in the survey, older adults might interpret putting off care differently. Although we reported the type of care older adults put off during the pandemic, information about the severity of care is not available. For example, a specialist appointment with a cardiac consultant for an older person with unstable angina would be a lot more critical than an annual vision examination appointment. Fifth, we used the physical frailty phenotype proposed by Fried and colleagues [4]. However, participants’ decisions on whether the risk of getting infected outweighed the risk of not seeking care may be more likely to have been based on the information they had received about these ‘vulnerable groups’ than on their general physical frailty. To address this, we controlled the number of high-risk conditions for COVID-19 complications in our data analysis. Lastly, we discussed low health literacy may be one of the barriers to access to care. On the other hand, even people with high health literacy and good resources may be confronted with a dilemma: they knew the importance of continuity of care, but also the danger of exposing themselves to COVID. One should also take into account the high levels of disinformation and the political polarization of COVID-related issues. These issues were outside the scope of the study.

## 5. Conclusions

Overall, approximately two out of five older people in the survey reported postponing care as a result of the COVID-19 pandemic. Contrary to our hypotheses, in the NHATS COVID-19 sample as a whole frail people were no more likely to put off care than were pre-frail and robust people; and non-White people were no more likely than White people to put off care. However, these findings masked important differences, where frail non-White people appeared to be differentially affected by the pandemic. When comparing White and non-White people by their frailty status, we found significantly less putting off care among robust non-White people, and significantly more putting off care among frail non-White people. Our findings support the double jeopardy hypothesis. That is, racial/ethnic minority older adults suffer a double disadvantage in access to health care during the pandemic due to the interactive effects of frailty and race. However, these differences were hard to account for with our data. Non-White older people of all frailty statuses, were no more likely than White people to mention reasons for putting off care that would be indicative of systemic or personal barriers, such as a provider cancelled, closed, or suggested rescheduling, not getting an appointment, deciding it could wait, being afraid to go, and a family member not wanting them to go. These issues require further research with better measures of systematic racism and barriers to care and a sample that has larger and more inclusive minority group representation.

## Figures and Tables

**Table 1:** Characteristics of study participants (sample *n* = 2628; population *n* = 22,164,441).

	Percent or Mean	95% CI	
		Lower	Upper
Age (years)			
65–79	72.62%	71.03%	74.15%
80+	27.38%	25.85%	28.97%
Sex			
Male	45.96%	43.40%	48.54%
Female	54.04%	51.46%	56.60%
Race/ethnicity			
White, non-Hispanic	85.38%	82.93%	87.53%
Black, non-Hispanic	6.03%	5.10%	7.13%
Other	8.59%	6.71%	10.92%
Education level			
<High school	10.07%	8.83%	11.48%
≥High school	54.20%	5.11%	57.26%
≥Bachelor’s degree	35.73%	32.27%	39.34%
Marital status			
Unmarried	40.49%	37.93%	43.10%
Married/Partnered	59.51%	56.90%	62.07%
Location			
Metropolitan	80.94%	72.71%	87.13%
Non-metropolitan	19.06%	12.87%	27.29%
Number of chronic conditions	1.51	1.46	1.56
≤2 conditions	81.76%	79.97%	83.42%
3+ conditions	18.24%	16.58%	20.03%
Heart attack/myocardial infarction	1.32%	0.93%	1.87%
Heart disease	21.04%	19.10%	23.12%
High blood pressure	69.58%	67.14%	71.91%
Diabetes	26.48%	24.86%	28.17%
Lung disease	21.54%	19.86%	23.32%
Stroke	1.58%	1.09%	2.29%
Dementia or Alzheimer’s disease	2.99%	2.30%	3.88%
Cancer	6.63%	5.63%	7.81%
Others help with medicines			
Yes	13.32%	11.88%	14.91%
No	86.68%	85.09%	88.12%
Frailty			
Robust	14.93%	13.08%	16.99%
Pre-frail	70.99%	68.80%	73.10%
Frail	14.08%	12.56%	15.75%
Medical care in 2019			
Had regular doctor	96.78%	95.90%	97.47%
Saw the doctor *	94.99%	94.01%	95.81%
Put off care during COVID-19 pandemic			
Yes	40.63%	38.21%	43.09%
No	59.37%	56.91%	61.79%

Notes: Unmarried: separated/divorced/widowed/never married; CI: confidence interval. * sample *n* = 2623; population *n* = 22,141,941.

**Table 2:** Percentage of putting off care by frailty and race/ethnicity.

	Percent	95% CI		Sample *n*	Population *n*
		Lower	Upper		
**All**	40.63%	38.21%	43.09%	2628	22,164,441
**All:** Robust	37.91%	31.85%	44.37%	397	3,309,062
**All:** Pre-frail	41.05%	38.29%	43.87%	1802	15,735,313
**All:** Frail	41.38%	35.94%	47.04%	429	3,120,066
**All:** White, non-Hispanic	41.03%	38.40%	43.70%	2055	18,924,178
**All:** Black, non-Hispanic/other	38.31%	33.09%	43.82%	573	3,240,263
**White, non-Hispanic:** Robust	39.87%	33.34%	46.79%	298	2,731,161
**White, non-Hispanic:** Pre-frail	41.55%	38.52%	44.65%	1434	13,599,045
**White, non-Hispanic:** Frail	39.48%	34.13%	45.10%	323	2,593,972
**Black, non-Hispanic/other:** Robust	28.64%	18.12%	42.12%	99	577,901
**Black, non-Hispanic/other:** Pre-frail	37.88%	30.84%	45.47%	368	2,136,267
**Black, non-Hispanic/other:** Frail	50.70%	39.23%	62.10%	106	626,095

Note: CI: confidence interval.

**Table 3:** Logistic regression results for putting off care.

Outcome: Putting off Care	OR	*p* Value	95% CI	
			Lower	Upper
Frailty (ref: robust)				
Pre-frail	1.066	0.649	0.806	1.411
Frail	1.154	0.467	0.780	1.706
Race/ethnicity (ref: White, non-Hispanic)				
Black, non-Hispanic/other	0.630	0.127	0.347	1.145
Frailty * Race/ethnicity				
Pre-frail, Black, non-Hispanic/other	1.483	0.285	0.714	3.084
Frail, Black, non-Hispanic/other	**2.688**	**0.009**	1.300	5.556
Age (ref. <80 years old)				
80+	0.865	0.212	0.688	1.088
Female	**1.724**	**<0.001**	1.429	2.079
Marital status (ref: unmarried)				
Married/partnered	1.097	0.483	0.844	1.426
Education (ref: <high school)				
High school, associate’s degree	1.240	0.242	0.861	1.785
Bachelor’s degree or above	**2.299**	**<0.001**	1.477	3.578
Location (ref: non-metropolitan)				
Metropolitan	**1.254**	**0.047**	1.003	1.567
COVID comorbid conditions (ref: <3)				
3+ conditions	0.982	0.881	0.766	1.257
Others help with medicines (ref: no)				
Yes	0.963	0.817	0.695	1.333

Notes: OR: odds ratio; SE: standard error; CI: confidence interval. Boldface indicates statistical significance (*p* < 0.05). Frailty * Race/ethnicity indicates the interaction term between frailty and race/ethnicity.

**Table 4:** Reasons for putting off care by frailty and race/ethnicity.

Reasons for Putting off Care	All (Sample *n* = 1027; Population *n* = 9,005,379)	Robust (Sample *n* = 137; Population *n* = 1,254,495)		Pre-Frail (Sample *n* = 710; Population *n* = 6,459,901)		Frail (Sample *n* = 180; Population *n* = 1,290,983)		*p* Value
		White	Non-White	White	Non-White	White	Non-White	
		(*n* = 106)	(*n* = 31)	(*n* = 574)	(*n* = 136)	(*n* = 131)	(*n* = 49)	
The provider cancelled, closed, or suggested rescheduling	56.91%	57.42%	53.51%	58.94%	50.03%	53.54%	47.68%	0.391
I decided it could wait	52.12%	50.17%	56.01%	53.70%	48.33%	53.78%	29.29%	0.453
I was afraid to go	28.05%	24.31%	44.24%	28.15%	29.05%	29.62%	22.07%	0.728
I couldn’t get an appointment	10.55%	15.09%	0%	10.08%	13.06%	8.48%	8.86%	0.589
A family member did not want me to go	8.15%	9.04%	5.96%	7.77%	9.66%	9.60%	3.74%	0.374
I couldn’t afford it	2.30%	6.78%	5.95%	1.28%	4.28%	0.65%	3.81%	0.189
In quarantine	0.39%	0%	0%	0.25%	0%	1.91%	0.60%	0.326
Other	3.77%	2.65%	0%	3.86%	4.01%	2.12%	14.54%	0.550

## Data Availability

The National Health and Aging Trends Study (NHATS) is publicly available at https://www.nhats.org/researcher, (accessed on 6 January 2022).

## Supplementary Materials

The following supporting information can be downloaded at: https://www.mdpi.com/article/10.3390/ijerph20021034/s1, Table S1: Pre-pandemic health care activities in Round 9.
