# Supplementary material for: Are Frail Older People from Racial/Ethnic Minorities at Double Jeopardy of Putting off Healthcare during the Pandemic?
Source: Int J Environ Res Public Health. 2023 Jan 6;20(2):1034. doi: 10.3390/ijerph20021034 (PMC9859101; doi:10.3390/ijerph20021034)
Supplement: Supplementary file 1 [file ijerph-20-01034-s001.zip › ijerph-1895988-supplementary.pdf]

Supplemental Material

Table S1. Pre-pandemic health care activities in Round 9

Questions	Overall		Frailty				Race/Ethnicity		
	**N**	**Percentage**	**Robust**	**Pre-Frail**	**Frail**	**P Value**	**White**	**Non-White**	**P Value**
In the last year, how did you get to your regular doctor/the doctor?									
Drove yourself	2490	81.18%	82.87%	85.27%	59.64%	**<0.001**	85.06%	59.20%	**<0.001**
A ride from a family member or friend	2490	21.34%	19.37%	17.11%	43.84%	**<0.001**	19.01%	34.53%	**<0.001**
In the last year, did anyone sit in with you and your regular doctor during your visits?	2490	33.33%	22.13%	31.38%	54.18%	**<0.001**	32.34%	38.97%	0.920
Help you with getting on the exam table, dressing, and undressing?	932	9.64%	4.79%	5.20%	24.11%	**<0.001**	8.53%	14.87%	**<0.001**
Remind you about things you wanted to ask or tell the doctor?	932	57.66%	45.51%	56.29%	66.57%	**<0.001**	59.03%	51.22%	0.500
Ask or tell the doctor things for you?	929	50.71%	40.78%	46.70%	66.06%	**<0.001**	50.06%	53.76%	**0.010**
Help you understand what the doctor was saying?	932	46.81%	39.19%	43.44%	59.44%	**<0.001**	45.14%	54.66%	**0.012**
